# Mental health of divorcees through divorce by mutual consent in Japan

**DOI:** 10.1002/pcn5.229

**Published:** 2024-07-29

**Authors:** Reiko Otaki, Yuka Igarashi, Yotaro Katsumata

**Affiliations:** ^1^ Tokyo Metropolitan, University Tokyo Japan

In Japan, the annual number of divorces reached 210,000 in 2020, with approximately 90% of them opting for mutual consent divorce, which is finalized solely upon the agreement of the couples involved. The negative impact of divorce on mental health has long been reported,^1^ and it is well known that it increases the risk of suicide.^2^ Unlike many other countries, divorces in Japan are often concluded without the intervention of the judiciary or other professionals. In Japan, while the correlation between divorce and suicide rates has been examined utilizing macro data,^3^ there have been limited studies on the mental health issues faced by divorced individuals, the contributing factors, and the available support systems. To address this gap, the Japanese Ministry of Justice conducted a web‐based survey^4^ in March 2021 to ascertain the status of mutual consent divorces. The survey targeted 1000 parents with minor‐aged children who had experienced mutual consent divorce in the past decade and were in their 30s or 40s across Japan.

This study aims to examine the actual conditions and associated factors of mental health among individuals who have undergone mutual consent divorce, utilizing data obtained from the Ministry of Justice survey. Data from 244 individuals (118 men and 126 women) who had undergone divorce between 2016 and 2021, and had completed the K6 mental health screening^5^, ^6^ reflecting on the immediate aftermath of their separation, were included in the analysis (Figure 1). As over 80% of Japanese couples who opt for mutual consent divorce do so after a period of separation, this study specifically focuses on individuals who have undergone a typical divorce process in Japan.

**Figure 1:**
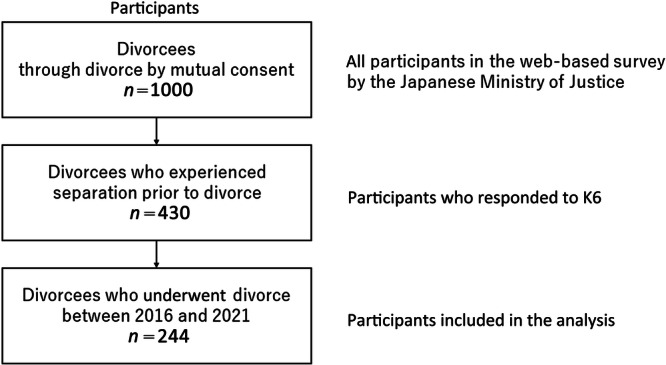
Process for selecting participants for analysis in this study.

This analysis revealed that the average K6 score immediately after separation was 11.35 (SD = 3.11), with approximately 45% of participants above 13, indicative of severe mental disorders. Additionally, a *χ* ^2^ test was used to examine the factors associated with the K6 severity category (13 points or more), and the results showed that those who used a public consultation before separation were significantly more likely to fall into the severe category of K6 scores (*p* = 0.02). Other factors, such as education, income, preseparation discussions, and arrangements, were not significantly associated with the severity of K6 scores. In comparison, the general population in Japan reported a 4.6% prevalence of scores of 13 or higher and 25.8% for scores of 5–12,^7^ indicating poorer mental health among those who had undergone mutual consent divorce. The reasons behind the poor mental health among individuals who sought consultation before separation are not fully understood, but it is possible that complex factors, such as domestic violence, may contribute to this phenomenon.

This study is based on the secondary analysis of the Ministry of Justice survey data, and the K6 responses reflect scores obtained retrospectively through reflection on the period immediately after separation. Consequently, the details of life stage changes associated with divorce remain unclear. Nevertheless, even when compared with the distribution of K6 scores in the general population of Japan, the findings of this study, with approximately 45% of individuals experiencing severe mental distress, underscore the critical need for accessible legal and mental health support services for those undergoing mutual consent divorce. Since the survey conducted by the Ministry of Justice was conducted online, self‐selection bias and sample representativeness issues remain a limitation of this analysis. In the future, it is imperative to explore the relationship between custody rights and the extent of disputes, and to develop support systems that prioritize the mental well‐being of parents and the healthy development of children.

## AUTHOR CONTRIBUTIONS

All authors have been personally and actively involved in substantive work leading to the report, and will hold themselves jointly and individually responsible for its content. All authors have seen and approved the manuscript and contributed significantly to the work.

## CONFLICT OF INTEREST STATEMENT

The authors declare no conflict of interest.

## ETHICS APPROVAL STATEMENT

This study was approved by the Ethics Committee of Tokyo Metropolitan University (Receipt Number: H5‐191).

## PATIENT CONSENT STATEMENT

Not applicable.

## CLINICAL TRIAL REGISTRATION

Not applicable.

## Data Availability

Not applicable.
